# CPD: Test yourself

**Published:** 2011-12

These continuing professional development (CPD) Test Yourself questions are based on the contents of this issue. You can use the questions to test your own understanding; we hope that you will also discuss them with your colleagues and other members of the eye care team. The questions have been developed in association with the International Council of Ophthalmology (ICO) and are based on the style of the ICO Advanced Examination: www.icoexams.org/exams/advanced

**Table:** 

**1.**	**Think about purchasing instruments and consumables. Which of the following statements are true, and which are false?**	**True**	**False**
a	Pre-shipment inspections are expensive, but they are worth the cost.	□	□
b	Your supplier is usually responsible for inland transportation.	□	□
c	When obtaining a quote, always approach more than one supplier.	□	□
d	The Incoterm® CIP means that you are responsible for insurance and clearance charges.	□	□
**2.**	**Think about instrument care and maintenance. Which of the following statements are true, and which are false?**	**True**	**False**
a	Instruments must be cleaned after each use and lubricated once a week.	□	□
b	Using distilled water to clean instruments reduces the risk of corrosion and chemical damage.	□	□
c	Regular inspection is important, but it is not necessary to do so under magnification.	□	□
d	It is important to keep records of any instruments requiring repair or replacement.	□	□
**3.**	**Think about supplies management. Which of the following statements are true, and which are false?**	**True**	**False**
a	You don't have to make a large order to take advantage of bulk discounts.	□	□
b	The people who manage supplies are usually specially trained and enjoy working with complex inventory systems.	□	□
c	If an eye unit uses between 20 and 30 sutures a month, and they take between 6 and 8 weeks to arrive, you should keep a minimum 40 sutures in stock (and order more whenever the stock reaches 40).	□	□
d	The ‘first in, first out’ approach means that you must use the items with the shortest shelf life first.	□	□
**4.**	**Think about performing Schirmer's test and about sharpening and adjusting surgical scissors. Which of the following are true and which are false?**	**True**	**False**
a	When performing Schirmer's test, patients must close their eyes as tightly as possible.	□	□
b	Performing Schirmer's test soon after instilling any eyedrops will give a false result.	□	□
c	When sharpening surgical scissors, don't be afraid to apply pressure with the sharpening stone.	□	□
d	When sharpening surgical scissors, cover the full length of the cutting surface with each stroke.	□	□

## ANSWERS

1. **a. False.** If your country does not require one, only consider a pre-shipment inspection (PSI) if you are making a large order. A PSI may speed up clearance on arrival, but will increase the price by 2–3%. You should decide whether the cost is worth it. **b. False.** You are responsible, unless you have chosen for the items to be couriered to you (i.e., door-to-door delivery), **c. True. d. False.** You are responsible for clearance, but marine insurance is the seller's responsibility.
2. **a. False.** Instruments should be cleaned and lubricated after each use. **b. True. c. False.** Use magnification wherever possible to help you identify instruments that must be repaired or serviced, **d. True**. This allows you to plan ahead so you can replace or repair instruments as needed.
3. **a. True**. You can negotiate a discount based on projected demand, **b. False.** Make sure your inventory systems easy and convenient to use. **c. False.** Stock may take as long as 8 weeks to arrive (around 2 months), and you may use as much as 30 sutures a month, so keep enough to last until then (2 × 30 = 60). **d. True.**
4. **a. False.** Patients must close their eyes as lightly as possible, **b. True. c. False.** Do not apply too much force. The repetition of the movement is what sharpens the scissors, **d. True.**

